# Differentiation of Brain Abscesses from Glioblastomas and Metastatic Brain Tumors: Comparisons of Diagnostic Performance of Dynamic Susceptibility Contrast-Enhanced Perfusion MR Imaging before and after Mathematic Contrast Leakage Correction

**DOI:** 10.1371/journal.pone.0109172

**Published:** 2014-10-17

**Authors:** Cheng Hong Toh, Kuo-Chen Wei, Chen-Nen Chang, Shu-Hang Ng, Ho-Fai Wong, Ching-Po Lin

**Affiliations:** 1 Departments of Medical Imaging and Intervention, Department of Neurosurgery, Chang Gung Memorial Hospital, Linkou and Chang Gung University College of Medicine, Tao-Yuan, Taiwan; 2 Department of Neurosurgery, Chang Gung Memorial Hospital, Linkou and Chang Gung University College of Medicine, Tao-Yuan, Taiwan; 3 Department of Biomedical Imaging and Radiological Sciences, Institute of Neuroscience, National Yang-Ming University, Taipei, Taiwan; 4 Brain Connectivity Laboratory, Institute of Neuroscience, National Yang-Ming University, Taipei, Taiwan; The George Washington University, United States of America

## Abstract

**Purpose:**

To compare the diagnostic performance of dynamic susceptibility contrast-enhanced perfusion MRI before and after mathematic contrast leakage correction in differentiating pyogenic brain abscesses from glioblastomas and/or metastatic brain tumors.

**Materials and Methods:**

Cerebral blood volume (CBV), leakage-corrected CBV and leakage coefficient K_2_ were measured in enhancing rims, perifocal edema and contralateral normal appearing white matter (NAWM) of 17 abscesses, 19 glioblastomas and 20 metastases, respectively. The CBV and corrected CBV were normalized by dividing the values in the enhancing rims or edema to those of contralateral NAWM. For each study group, a paired t test was used to compare the K_2_ of the enhancing rims or edema with those of NAWM, as well as between CBV and corrected CBV of the enhancing rims or edema. ANOVA was used to compare CBV, corrected CBV and K_2_ among three lesion types. The diagnostic performance of CBV and corrected CBV was assessed with receiver operating characteristic (ROC) curve analysis.

**Results:**

The CBV and correction CBV of enhancing rim were 1.45±1.17 and 1.97±1.01 for abscesses, 3.85±2.19 and 4.39±2.33 for glioblastomas, and 2.39±0.90 and 2.97±0.78 for metastases, respectively. The CBV and corrected CBV in the enhancing rim of abscesses were significantly lower than those of glioblastomas and metastases (P = 0.001 and P = 0.007, respectively). In differentiating abscesses from glioblastomas and metastases, the AUC values of corrected CBV (0.822) were slightly higher than those of CBV (0.792).

**Conclusions:**

Mathematic leakage correction slightly increases the diagnostic performance of CBV in differentiating pyogenic abscesses from necrotic glioblastomas and cystic metastases. Clinically, DSC perfusion MRI may not need mathematic leakage correction in differentiating abscesses from glioblastomas and/or metastases.

## Introduction

It may be difficult to differentiate pyogenic brain abscesses from necrotic glioblastomas and cystic metastases with conventional MRI because all of them can appear as rim-enhancing masses with prominent perifocal edema [1]–[3]. Diffusion-weighted imaging (DWI) and diffusion-tensor imaging (DTI) that measures water diffusivity complement the role of conventional MR in the differentiation of abscesses from necrotic glioblastomas and metastases [1]. Mean water diffusivity, quantified with apparent diffusion coefficient (ADC), is typically low in abscess cavities but high in tumor cysts [1], [4]. However, high diffusivity similar to that found in necrotic tumors has been reported in 5–21% of untreated abscesses [5], [6]. On the other hand, necrotic glioblastomas [5], [7], [8] and cystic metastases [9]–[11] may demonstrate low diffusivity mimicking abscesses. In short, differentiation of brain abscesses from necrotic glioblastomas and cystic metastases based solely on water diffusion properties can sometimes be difficult.

Dynamic susceptibility contrast-enhanced (DSC) perfusion MRI has been used to differentiate abscesses from glioblastomas and/or metastases in a few preliminary studies [12]–[16]. DSC perfusion MRI is an advanced MR technique that measures T2*-weighted signal intensity loss, which occurs dynamically over bolus injection of contrast medium, and from which relative cerebral blood volume (CBV), a marker of angiogenesis can be computed [17]. On DSC perfusion MRI, the enhancing rims of abscesses typically demonstrate CBV that is lower than those of glioblastomas and metastases.

In lesions, particularly brain tumors with substantial blood-brain barrier breakdown and contrast leakage, the T2*-weighted signal intensity loss can be masked by signal intensity increase secondary to T1 effects. In such instances, CBV will be underestimated [17], [18]. To measure CBV with higher accuracy, a mathematic leakage-correction model has been proposed to process the DSC perfusion data [18]. It allows simultaneous assessment of tumor vascularity and permeability by calculating leakage-corrected CBV and the leakage coefficient K_2_, respectively. In high-grade gliomas, the corrected CBV were found to have better correlation with tumor grade than the uncorrected one [18]. The K_2_, on the other hand, was able to demonstrate differences in permeability among gliomas of different histologic grades [19].

The newly formed capillaries in abscess capsules lack tight junctions are also associated with increased vascular permeability [20]. As the DSC perfusion MRI reported in previous studies was not corrected for contrast leakage, comparisons of perfusion characteristics between abscesses and glioblastomas or metastases were therefore based on CBV that was theoretically underestimated. Although precise measurement is essential for CBV to serve as a quantitative criterion for clinical decision making, it requires extra time and sophisticated software packages for data postprocessing. Before implementing leakage correction as a routine, it is essential to investigate if it improves the diagnostic performance of CBV. In this study, we aimed to compare the diagnostic performance of DSC perfusion MRI before and after mathematic leakage correction in the differentiation of pyogenic brain abscesses from necrotic glioblastomas and cystic metastases.

## Materials and Methods

### Ethics Statement

Approval for this study was obtained from the Institutional Review Board of Chang Gung Memorial Hospital, Linkou, Taiwan. Written informed consent was obtained from all patients before imaging acquisition.

### Patients

Preoperative conventional and DSC perfusion MRI were performed in patients who were suspected of having pyogenic brain abscesses, necrotic glioblastomas or cystic metastatic brain tumors following prior CT or MRI examination. Patients were excluded if their estimated glomerular filtration rate, calculated from serum creatinine levels, patient demographics, and age, was less than 60 mL/min/1.73 m^2.^

All lesions appeared as a rim enhancing mass with perifocal edema on MRI. Images from 2 patients with pyogenic abscesses were excluded because of substantial motion artifact. Six glioblastomas and 5 metastases with large areas of hemorrhages preventing perfusion analysis were excluded. Included in the study were 17 patients (12 men, 5 women; mean age, 54.8 years; age range, 25–74 years) with pyogenic brain abscesses (mean size, 3.9 cm±0.9), 19 patients (14 men, 5 women; mean age, 59.8 years; age range, 27–79 years) with necrotic glioblastomas (mean size, 5.1 cm±1.1) and 20 (10 men, 10 women; mean age, 56.8 years; age range, 37–78 years) patients with solitary cystic metastatic brain tumors (mean size, 3.5 cm±1.3). The diagnosis of brain abscess was confirmed by surgery in all patients. Histological diagnosis was obtained in all patients with glioblastomas and metastases. In patients with brain metastases, primary tumors included lung carcinomas (n = 12), gastrointestinal carcinomas (n = 2), breast cancers (n = 4) and those from unknown primary site (n = 2). No patients had begun corticosteroid or antibiotics treatment at the time of their MR imaging.

### MRI

All MR studies were performed using a 3T unit (Magnetom Tim Trio, Siemens, Erlangen, Germany) equipped with a 12-channel phased-array head coil. Routine MR pulse sequences including transverse T1WI, transverse T2WI and transverse FLAIR. The DSC perfusion MRI was obtained with a T2*- weighted gradient-echo EPI sequence during the bolus injection of standard dose (0.1 mmol/kg) of intravenous gadopentetate dimeglumine (Magnevist; Schering, Berlin, Germany). The injection rate was 4 mL/s for all patients and was immediately followed by a bolus injection of saline (total of 20 mL at the same rate). DSC perfusion MRI sequence parameters included the following: TR/TE, 1640/40 ms; flip angle, 90°; FOV, 230230 mm; section thickness, 4 mm; 20 sections and acquisition time of 1 minute 30 seconds. Fifty measurements were acquired allowing acquisition of about 10 measurements before bolus arrival. The total time for image collection before, during, and after bolus contrast was about 25, 25 and 40 seconds, respectively. Preload contrast medium was not given before DSC perfusion MR imaging. Postcontrast 3D magnetization prepared rapid acquisition gradient echo (MPRAGE) images (TR/TE, 2000/2.63 ms; section thickness, 1 mm; inversion time, 900 ms; acquisition matrix, 224×256 and FOV, 224×256 mm) were acquired after completion of the DSC sequence.

### Image Postprocessing

The DSC data were transferred to an independent workstation and processed by using the software nordicICE (nordic Image Control and Evaluation Version 2.3, Nordic Imaging Lab, Bergen, Norway). For each voxel, the dynamic signal intensity curve was converted to a relaxivity-time curve (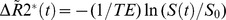, a parameter related to the concentration of gadolinium in the voxel. The CBV was estimated by integrating the relaxivity-time curve as described previously [17]. Contrast leakage correction was performed on the DSC images by using a technique outlined by Boxerman et al [18]. This method assumes that T1 shortening resulting from contrast leakage occurs in regions with disrupted BBB and uses linear fitting to determine the leakage coefficient K_2_, a first order estimate of vascular permeability proportional to the leakage, the product of permeability and surface area. The K_2_ was subsequently used for leakage correction and calculation of corrected CBV. The relationship between corrected CBV, uncorrected CBV and first-order estimate of vascular permeability is shown in the following equation:
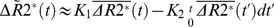



The CBV is calculated by trapezoidal integration of 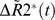. 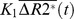 that reflects the corrected CBV and the K_2_ term reflects the effects of leakage. The K_1_ and K_2_ were determined by using a linear least squares fit to the equation.

### Image Analysis

The CBV, corrected CBV and K_2_ maps were coregistered to postcontrast MPRAGE based on 3D non-rigid transformation and mutual information with the use of Statistical Parametric Mapping 2 (Wellcome Department of Cognitive Neurology, London, UK) before all imaging analysis. Based on T2WI and postcontrast MPRAGE, the enhancing rim and the perifocal edema adjacent to the enhancing rim (approximately 5 mm width) of each lesion were manually segmented.

A polygonal region-of-interest (ROI) was drawn to include entire enhancing lesion. First, the enhancing rim and then the perifocal edema were manually segmented by scatter ROIs created with pixel thresholding. The steps were repeated slice by slice to include the entire lesion. The segmented ROIs were subsequently used to measure the mean values of CBV, corrected CBV and K_2_ of entire lesion, respectively. K_2_ is the leakage coefficient that is proportional to the leakage, the product of permeability and surface area [18]. All ROIs did not include areas of necrosis or nontumor macrovessels evident on postcontrast MPRAGE and T2WI.

For all quantitative comparisons, CBV and corrected CBV were normalized and displayed as a ratio by dividing their values in either the enhancing rim or edema by the values in the contralateral normal-appearing white mater (NAWM). The K_2_ values of enhancing rim or edema were not normalized as those of NAWM were equal or close to zero in most cases.

### Statistical Analysis

For each study group, a paired t test was used to compare between K_2_ values of enhancing rim or edema and their corresponding NAWM, as well as the CBV and corrected CBV of the enhancing rim and edema. Comparisons among abscesses, glioblastomas and metastases for the mean values of CBV, corrected CBV and K_2_ measured in the enhancing rim or edema respectively, were performed using ANOVA. Multiple pairwise comparisons were performed with the Games-Howell method.

Cutoff values of CBV, corrected CBV and K_2_ for distinguishing abscesses from glioblastomas and/or metastases were determined by receiver operating characteristic (ROC) curve analysis. The values of area under the curve (AUC), measurements of overall diagnostic performances, were calculated from the ROC curves. SPSS 18.0 (SPSS, Chicago, Illinois) software was used to perform the statistical analyses, and P values<.05 were considered to indicate a statistically significant difference.

## Results

**Figure 1:**
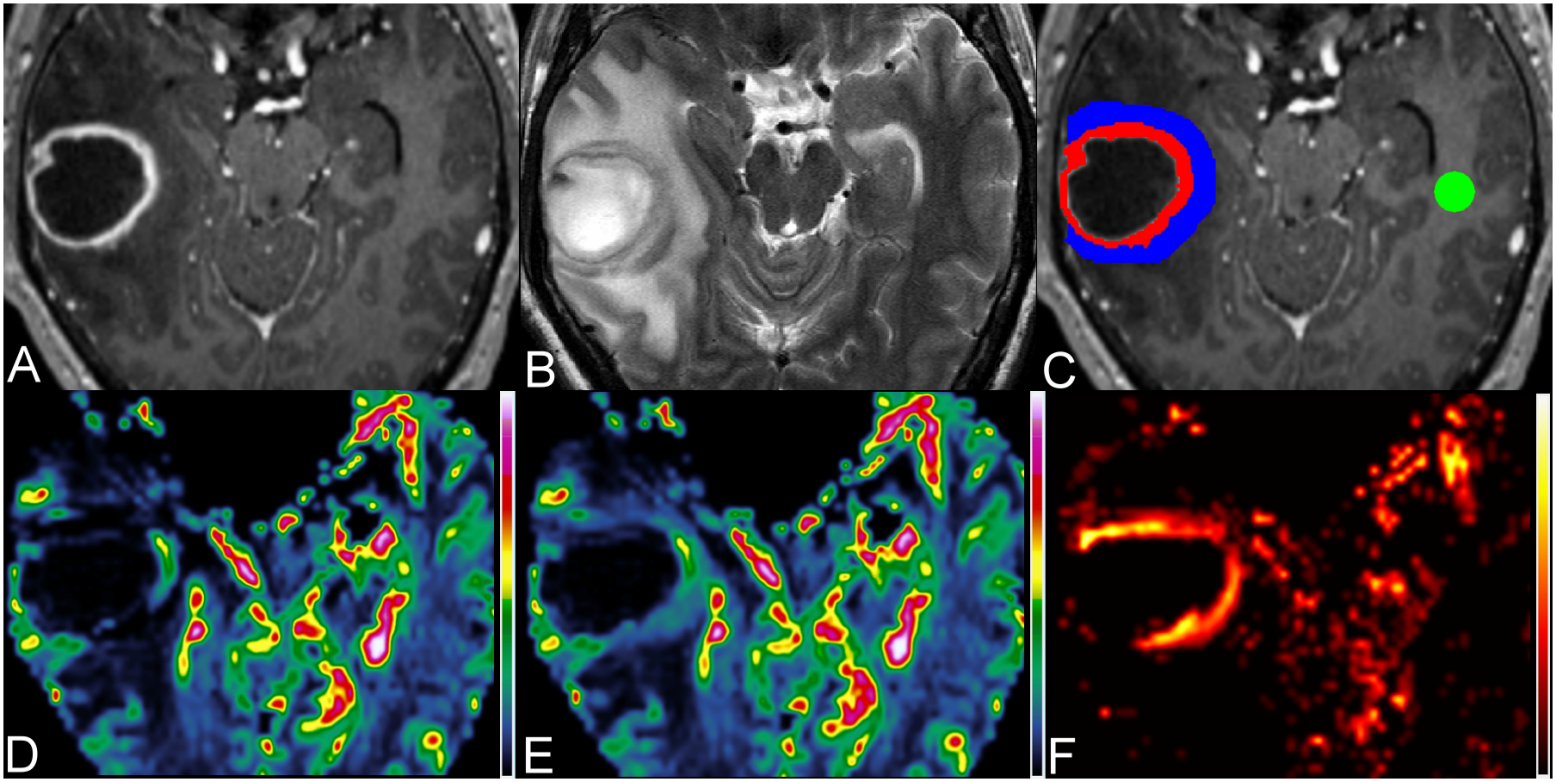
Measurements of perfusion parameters in a 47-year-old man with pyogenic brain abscess. Axial contrast-enhanced MPRAGE (A) and T2W image (B) show a rim-enhancing mass with perifocal edema in the right temporal lobe. (C) On contrast-enhanced MPRAGE, three ROIs are placed over the enhancing rim (red), perifocal edema (blue) most adjacent to the enhancing rim and the contralateral NAWM (green) for the measurements of CBV (D), corrected CBV (E) and K_2_ (F), respectively.

**Figure 2:**
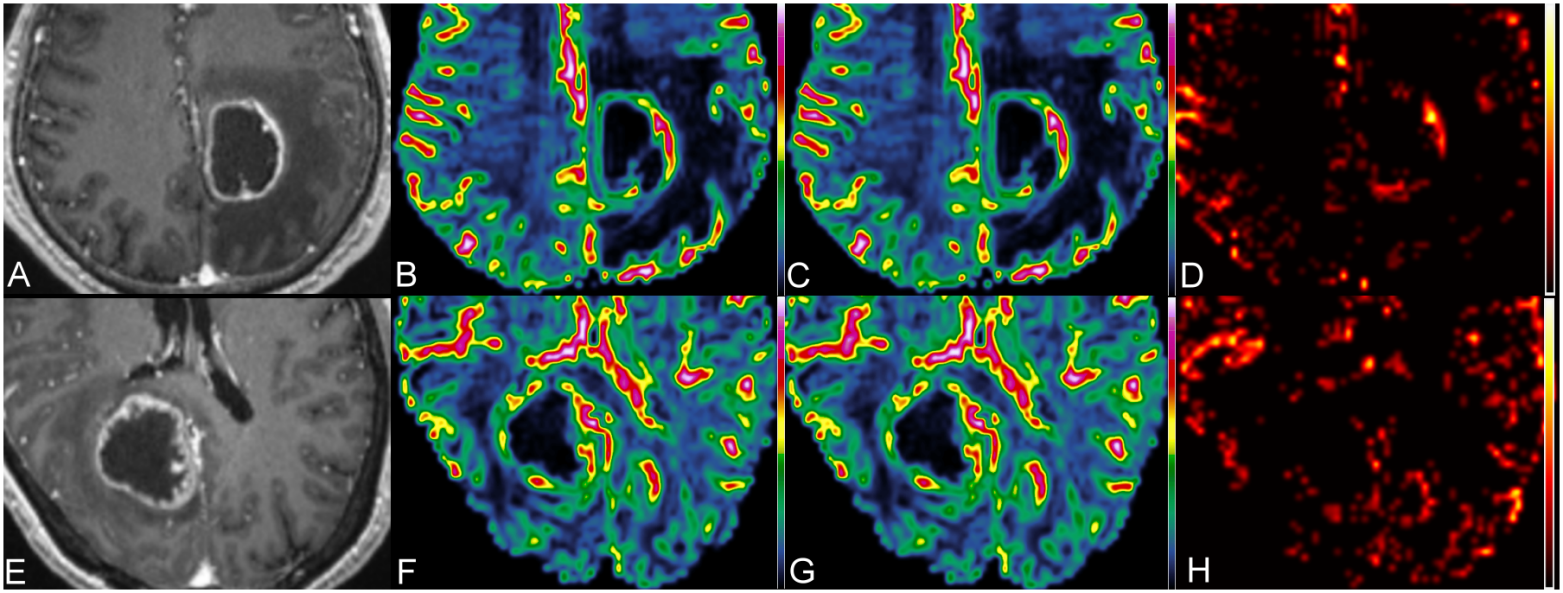
MR perfusion of a glioblastoma and a metastatic brain tumor. The upper panel shows the contrast-enhanced MPRAGE (A), CBV (B), corrected CBV (C) and K_2_ (D) images of a necrotic glioblastoma in the left medial parietal region, and the lower panel (E, F, G and H) shows the corresponding images of a cystic metastatic brain tumor in the right occipital lobe.

Figure 1 illustrates the measurements of perfusion parameters in a pyogenic brain abscess. Figure 2 demonstrates perfusion images of a necrotic glioblastoma and a metastatic brain tumor, respectively. The mean K_2_ values of enhancing rim, edema and NAWM were 1.33±1.29, 0.28±0.27 and 0.01±0.02, respectively, for abscesses, were 0.92±0.79, 0.14±0.16 and 0.01±0.05, respectively, for glioblastomas, and were 1.22±1.45, 0.11±0.10 and 0.05±0.15, respectively, for metastases. Abscesses and glioblastomas demonstrated significant K_2_ increases in their enhancing rims and edema as compared with the corresponding NAWM. For metastases, the increase of K_2_ was significant in the enhancing rim (P = 0.001) but not in the edema (P = 0.135).

The mean values of CBV and corrected CBV were 1.45±1.17 and 1.97±1.01, respectively, for abscesses, were 3.85±2.19 and 4.39±2.33, respectively for glioblastomas, and were 2.39±0.90 and 2.97±0.78, respectively for metastases. The differences between CBV and corrected CBV in the enhancing rim were significant for abscesses (P<.001), glioblastomas (P<.001) and metastases (P = 0.002). In the edema, the differences between CBV and corrected CBV were significant for abscesses (P<.001) and glioblastomas (P = 0.001), but not for metastases (P = 0.557).

**Figure 3:**
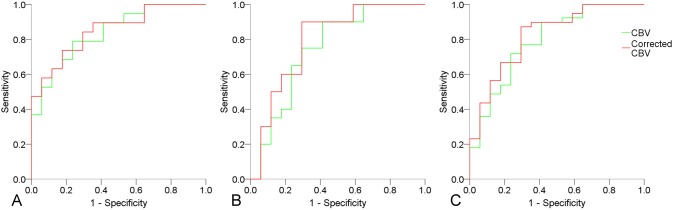
ROC curve analysis of CBV and corrected CBV in differentiating abscesses from glioblastomas (A), abscesses from metastases (B), and abscesses from glioblastomas and metastases.

Table 1 shows the results of quantitative comparisons of the perfusion parameters among the 3 study groups. The K_2_ in the enhancing rim and edema showed no significant differences among the 3 study groups. The CBV and corrected CBV of enhancing rims were significantly lower in abscesses than in glioblastomas and metastases. The CBV and corrected CBV in the edema were not significantly different among the 3 groups. Table 2 summarizes the results of ROC analysis. In differentiating abscesses from glioblastomas and/or metastases, the AUC values of corrected CBV were higher than those of CBV. The results of ROC curve analysis comparing diagnostic performance of CBV and corrected CBV in differentiating abscesses from glioblastomas and/or metastases are illustrated in Figure 3.

**Table 1:** Quantitative comparisons of perfusion parameters among abscesses, glioblastomas and metastases.

Region	Parameter	Abscess	GB	Mets	Abscess vs. GB	Abscess vs. Mets	GB vs. Mets
Enhancing rim	K_2_	1.33±1.29	0.92±0.79	1.22±1.45	0.508	0.968	0.706
	CBV	1.45±1.17	3.85±2.19	2.39±0.90	0.001*	0.030*	0.034*
	Corrected CBV	1.97±1.01	4.39±2.33	2.97±0.78	0.001*	0.007*	0.048*
Perifocal edema	K_2_	0.28±0.27	0.14±0.16	0.11±0.10	0.198	0.072	0.786
	CBV	1.04±0.43	1.54±1.21	0.88±0.33	0.242	0.432	0.082
	Corrected CBV	1.21±0.40	1.62±1.24	0.90±0.35	0.380	0.053	0.063

Note. – Data are mean ± standard deviation; CBV and corrected CBV are in ratios to corresponding NAWM, GB, glioblastomas; Mets, metastasis; *p<0.05.

**Table 2:** ROC analysis of CBV and corrected CBV of enhancing rim in differentiating abscesses from glioblastomas and/or metastases.

Group	Parameter	AUC	95% CI	P value	CV	SEN	SPE	ACC
Abscess vs. GB	CBV	0.839	0.712–0.966	0.001*	2.17	78.9	76.5	77.8
	Corrected CBV	0.851	0.729–0.974	<.001*	2.75	73.7	82.4	77.8
Abscess vs. Mets	CBV	0.747	0.580–0.915	0.010*	1.53	90.0	58.8	75.7
	Corrected CBV	0.794	0.639–0.949	0.002*	2.05	90.0	70.6	81.1
Abscess vs. GB & Mets	CBV	0.792	0.658–0.925	0.001*	1.51	89.7	58.8	80.4
	Corrected CBV	0.822	0.700–0.944	<.001*	2.05	87.2	70.6	82.1

Note. – ACC, accuracy; CI, confidence interval; CV, cutoff value; GB, glioblastomas; Mets, metastasis; SEN, sensitivity; SPE, specificity; Data of sensitivity, specificity and accuracy are in percentage; *p<0.05.

## Discussion

In the present study, the enhancing rims of pyogenic abscesses demonstrated CBV that were significantly lower than those of glioblastomas and metastases, a finding that is in agreement with those reported in the literature [12]–[16]. At pathologic examination, the enhancing rims of abscesses represent fibrous capsules formed by collagen and are devoid of prominent neovascularization [21]–[23], whereas in glioblastomas and metastases the enhancing rims contain viable tumor cells. The microvessel density (MVD), a histologic measurement of angiogenesis, was found to be lower in abscesses when compared with high-grade gliomas [20]. In gliomas, there was a positive correlation between CBV and MVD [24]. Although the correlation has not been investigated with regard to abscesses and metastases, we speculate that the CBV differences among the 3 study groups reflect their MVD differences.

Although previous studies [12]–[16] had demonstrated the potential of DSC perfusion MRI in differentiating abscesses from glioblastomas and metastases, these were associated with some limitations. First, sample size was small and included only a few cases of pyogenic abscesses [12]–[15]. Second, coregistration between postcontrast T1WI and the perfusion maps was not performed in many studies [13]–[16]. Without proper image coregistration, it may be difficult to identify the exact location of the enhancing rim on CBV maps and thus, voxels from adjacent cystic cavity and perifocal edema may be included during ROI placement. Finally, most of the previous studies measured the perfusion parameters with one or a few small ROIs that were manually placed, and that is associated with inherent subjectivity. The last two may introduce significant errors in the measurement of perfusion parameters. In the present study, several steps were taken to overcome these limitations. Our relatively large sample size allowed the performance of DSC perfusion MRI in differentiating abscesses from glioblastomas and/or metastases to be evaluated more extensively. We also applied rigorous image registration and segmentation to enhance measurement accuracy and reduce subjectivity. Floriano et al. [25] recently reported good discriminatory ability of CBV, which has an AUC of 0.964, sensitivity of 97.8% and specificity of 92.6% in distinguishing between infectious and neoplastic brain lesions. As compared with their results, CBV in the present study has an AUC of 0.822, sensitivity of 87.2% and specificity of 70.6% in differentiating abscesses from glioblastomas and metastases. In their study, only 2 out of 46 infections were pyogenic abscesses. In contrast, all the infectious lesions included in the present study are pyogenic abscesses. Therefore, it seems that diagnostic performance of CBV in differentiating neoplasms from non-pyogenic infections is better than that in differentiating neoplasms from pyogenic infections.

The K_2_ values of the enhancing rims in all studied groups were higher than those of the corresponding NAWM, indicating that there was increased permeability in the enhancing rims. However, the permeability increase was not different among the 3 groups, suggesting that the changes of permeability in infections and neoplasms are similar. Angiogenesis is a complex multistep process with formation of immature vessels and changes of vascular permeability that can be seen in both tumoral and non-tumoral conditions. Increased vascularity may not be accompanied by increased permeability, and vice versa [26]. There was evidence suggesting that CBV and leakage coefficient may measure these two different aspects of angiogenesis [26]. In the present study, the enhancing rims of the 3 study groups demonstrated different CBV but similar K_2_, indicated that there may be different degrees of vascular hyperplasia but similar permeability in their enhancing rims.

In the past, comparisons of vascular permeability between pyogenic abscesses and glioblastomas and/or metastases has only been performed with perfusion CT using permeability surface area product [27] or dynamic contrast-enhanced (DCE) MRI [20] based on quantification of the volume transfer constant K^trans^. The results of these studies were not consistent. The permeability of abscesses was lower than those of glioblastomas and metastases on CT perfusion, but higher than those of high-grade gliomas on DCE MRI. Using DSC perfusion MRI, we found no differences in vascular permeability among the 3 study groups. This discrepancy may be due to different sample size, which was smaller in the previous two studies, and the different techniques used for assessment of vascular permeability.

Preload contrast medium administration and mathematic leakage correction are two different approaches to correct the T1 effects associated with contrast leakage. Several studies found that preload improves accuracy of CBV measurements in addition to leakage correction [28]–[30]. Boxerman et al [28] demonstrated that the two methods had synergistic effects and that combining the two improved accuracy and precision of CBV measurements. Hu et al [30] suggested that a 0.1-mmol/kg amount of preload gadodiamide plus a 6-minute delay time improved diagnostic accuracy of DSC perfusion MRI in treated gliomas. However, the authors also acknowledged that their protocol might not apply to other pathologies with different degrees of BBB disruption and local contrast diffusion rates.

Using mathematic leakage correction, we found that the diagnostic performance of corrected CBV was slightly better than the one without leakage correction. The increase of CBV in the enhancing rims of the 3 study groups following leakage correction may imply greater accuracy in CBV measurement. Preload contrast medium was not administered in the present study as there is no guideline or recommendation on the dose of preload contrast medium. Previous studies reported that K_2_ may correlate positively with K^trans^ with high flip angles [31], and that this correlation may disappear if a preload is used [32]. Therefore, to allow comparisons of both CBV and permeability among abscesses, glioblastomas and metastases, we chose not to have preload contrast medium administration in the present study.

In the present study, leakage correction increased specificity at the expense of slightly decreasing the sensitivity of CBV in differentiating among the 3 lesions. The overall diagnostic performance of CBV, measured with AUC derived from ROC curve analysis, improves slightly after leakage correction, particularly in differentiating between abscesses and glioblastomas. The improvement is more obvious when differentiating abscesses from metastases, or from metastases and glioblastomas. It is not known if the slight improvement is due to lower accuracy of CBV measurement as preload contrast medium was not given. Although higher accuracy in CBV measurement may potentially be associated with improved diagnostic performance [18], [30], decreased diagnostic performance after leakage correction has also been reported [33]. It has been reported that in differentiating between primary CNS lymphoma and glioblastoma, uncorrected CBV was better than corrected one [33].

The leakage correction method used in the present study is limited by its sensitivity to mean transit time (MTT) [34]. Elevation of MTT may cause CBV underestimation due to incorrect estimation of K_2_, and subsequently may affect CBV comparisons among the 3 study groups. Bjornerud et al [35], [36] proposed a novel postprocessing correction method which enables correction for both T_1_- and T_2_*-dominant leakage effects independent of MTT variations, and the use of their postprocessing correction schemes may improve the differentiation among abscesses, glioblastomas and metastases.

In conclusion, mathematic leakage correction slightly increases the diagnostic performance of CBV in differentiating pyogenic abscesses from necrotic glioblastomas and cystic metastases. It increases the specificity of CBV at the expense of decreasing sensitivity and additional postprocessing. Clinically, DSC perfusion MRI may not need mathematic leakage correction in differentiating abscesses from glioblastomas and/or metastases.
